# The Potential of Smartphone Apps in Informing Protobacco and Antitobacco Messaging Efforts Among Underserved Communities: Longitudinal Observational Study

**DOI:** 10.2196/17451

**Published:** 2020-07-07

**Authors:** Edmund WJ Lee, Mesfin Awoke Bekalu, Rachel McCloud, Donna Vallone, Monisha Arya, Nathaniel Osgood, Xiaoyan Li, Sara Minsky, Kasisomayajula Viswanath

**Affiliations:** 1 Dana-Farber Cancer Institute Boston, MA United States; 2 Harvard TH Chan School of Public Health Boston, MA United States; 3 Wee Kim Wee School of Communication and Information Nanyang Technological University Singapore Singapore; 4 Schroeder Institute Truth Initiative Washington, DC United States; 5 College of Global Public Health New York University New York, NY United States; 6 Department of Health, Behavior and Society Johns Hopkins University Bloomberg School of Public Health Baltimore, MD United States; 7 Baylor College of Medicine Houston, TX United States; 8 Center for Innovation in Quality, Effectiveness and Safety Michael E DeBakey VA Medical Center Houston, TX United States; 9 Department of Computer Science College of Arts and Science University of Saskatchewan Saskatoon, SK Canada

**Keywords:** mobile health, mobile phone, tobacco use, big data, spatial analysis, data science

## Abstract

**Background:**

People from underserved communities such as those from lower socioeconomic positions or racial and ethnic minority groups are often disproportionately targeted by the tobacco industry, through the relatively high levels of tobacco retail outlets (TROs) located in their neighborhood or protobacco marketing and promotional strategies. It is difficult to capture the smoking behaviors of individuals in actual locations as well as the extent of exposure to tobacco promotional efforts. With the high ownership of smartphones in the United States—when used alongside data sources on TRO locations—apps could potentially improve tobacco control efforts. Health apps could be used to assess individual-level exposure to tobacco marketing, particularly in relation to the locations of TROs as well as locations where they were most likely to smoke. To date, it remains unclear how health apps could be used practically by health promotion organizations to better reach underserved communities in their tobacco control efforts.

**Objective:**

This study aimed to demonstrate how smartphone apps could augment existing data on locations of TROs within underserved communities in Massachusetts and Texas to help inform tobacco control efforts.

**Methods:**

Data for this study were collected from 2 sources: (1) geolocations of TROs from the North American Industry Classification System 2016 and (2) 95 participants (aged 18 to 34 years) from underserved communities who resided in Massachusetts and Texas and took part in an 8-week study using location tracking on their smartphones. We analyzed the data using spatial autocorrelation, optimized hot spot analysis, and fitted power-law distribution to identify the TROs that attracted the most human traffic using mobility data.

**Results:**

Participants reported encountering protobacco messages mostly from store signs and displays and antitobacco messages predominantly through television. In Massachusetts, clusters of TROs (Dorchester Center and Jamaica Plain) and reported smoking behaviors (Dorchester Center, Roxbury Crossing, Lawrence) were found in economically disadvantaged neighborhoods. Despite the widespread distribution of TROs throughout the communities, participants overwhelmingly visited a relatively small number of TROs in Roxbury and Methuen. In Texas, clusters of TROs (Spring, Jersey Village, Bunker Hill Village, Sugar Land, and Missouri City) were found primarily in Houston, whereas clusters of reported smoking behaviors were concentrated in West University Place, Aldine, Jersey Village, Spring, and Baytown.

**Conclusions:**

Smartphone apps could be used to pair geolocation data with self-reported smoking behavior in order to gain a better understanding of how tobacco product marketing and promotion influence smoking behavior within vulnerable communities. Public health officials could take advantage of smartphone data collection capabilities to implement targeted tobacco control efforts in these strategic locations to reach underserved communities in their built environment.

## Introduction

### Background

Tobacco use is a major risk factor for lung cancer and premature morbidity [1] and also one of the leading preventable causes of death in the United States [2,3]. Approximately 480,000 deaths (1 in 5 deaths) in the United States annually could be attributed to smoking or tobacco consumption [4]. Although tobacco use is a prevalent public health problem in the United States, research has shown that the health and economic burden of tobacco use disproportionately affect underserved communities and that these communities have not benefitted from tobacco control efforts as much as others [5]. Research has also shown that the density of tobacco retail outlets (TROs) is higher in low-income neighborhoods [6] as well as in communities with higher percentages of ethnic minorities [7,8]. In addition, people from lower socioeconomic positions (SEPs) are often the target of the tobacco industry’s advertising, including the places of their residence [9]. The presence of TROs, together with a disproportionate exposure to protobacco messages, is associated with smoking behaviors and may attenuate the attempts of smokers to quit by allowing easy access to tobacco products as well as encouraging impulse purchases by providing environmental cues for smoking [10].

Although many studies have examined how factors such as the proximity of a residence to TROs and exposure to protobacco messages are related to smoking attitudes and initiation, smoking prevalence, and even hospital admissions [3,11-14], few studies have investigated the exposure and awareness of antitobacco messaging within underserved communities, particularly in relation to TROs and across media platforms. For instance, what are the media platforms where underserved communities are exposed to anti- or protobacco messages? Are people strategically exposed to antitobacco messages near locations such as TROs or places they are most likely to smoke or other places? To date, research on the extent of such antitobacco and protobacco message exposure is limited by its reliance on aggregated cross-sectional self-reported survey data.

### The Potential of Smartphone Apps in Informing Protobacco and Antitobacco Messaging Efforts

With the ubiquity of smartphone ownership, particularly within vulnerable communities, data from smartphone apps provide a potential data collection mechanism for informing health policy makers where to focus antitobacco messaging efforts [15,16], especially when they are used to complement traditional data sources such as the location of TROs. After all, smartphone penetration in the United States is high, with about 81% of the population owning a smartphone; the smartphone ownership figures are also high for underserved communities, such as people living in rural areas (71%), those making less than US $30,000 annually (71%), and in minority communities (approximately 79% to 80%) [17]. Health promotion organizations and policy makers could take advantage of the contextual information provided by smartphones to identify strategic areas to help ensure adequate exposure to antitobacco messages [18].

Smartphones enable researchers to *passively* engage in data collection at-scale within people’s naturalistic environments. For instance, by enabling geolocation tracking with the explicit consent of users, smartphone apps can collect temporally ordered information on the precise locations they visited or the paths they have taken, without being intrusive. The ability to track the behaviors of those in underserved communities in situ is a huge advantage over traditional survey methods that rely on self-reported recall [19]. Methods based on recall are limited in that participants may not remember all locations they have visited accurately, or they may omit details for the sake of social desirability (eg, underreporting of places they may perceive as undesirable). Geolocation tracking can provide valuable information for health organizations in helping to strategically target antitobacco messaging efforts by accurately identifying where protobacco messaging is being encountered. For example, the mobility or path data that outline how individuals move through their communities would be useful for identifying the most popular TROs and other place-based platforms where messages are being aired.

This type of smartphone data collection might also provide opportunities for health organizations to partner with underserved communities for *participatory science* efforts [20]. Although collecting data from the underserved communities can be extremely difficult [21], the use of smartphones could circumvent this problem as it adds a minimum logistical burden given the role of smartphones in their lives. The data collection could employ ecological momentary assessment (EMA) techniques, which assess particular events in the lives of subjects at periodic intervals, such as smoking behavior or exposure to specific types of messages, which are automatically prompted. These data, together with geolocation of smoking-related behaviors, could be used to map smoking *hot spots* [22]—defined as locations where there are non-random observed patterns of clustering—which are areas where there is a statistically significant clustering of respondents who report smoking in the same area.

These types of data collection efforts can facilitate the proactive reporting of exposure to tobacco messages that have high temporal specificity and are capable of capturing details of even ephemeral exposures (eg, a photo of an advertisement on a rotating billboard or as part of a video at a gas station and radio advertisements). Insights into where and how anti- and protobacco messages are reaching those in underserved communities can assist tobacco control practitioners and policy makers in helping reduce the disproportionate burden of tobacco use within vulnerable communities.

### Objectives of the Study

This study aimed to examine how smartphone app data collection could complement existing data sources to help inform tobacco control efforts for underserved communities. There are three specific objectives of this paper. First, we sought to identify if there were concentrations of TROs in Massachusetts and Texas. Second, using both passive (ie, geolocations) and active (ie, self-reports) data, we aimed to identify (1) the most popular TROs, denoted by a small number of TROs that attracted the most human traffic; (2) the areas in which participants were most likely to smoke; and (3) the locations where the participants reported exposure to tobacco messages and where the concentrations of pro- and antitobacco messages were. Third, we drew suggestions for tobacco control based on our results.

## Methods

### Study Design and Recruitment

To address the objectives of our study, we conducted a small-scale feasibility test using a smartphone app in underserved communities in Massachusetts and Texas. We have chosen to conduct the study in these 2 states, given the diversity in tobacco control policy implementation, where Massachusetts had stricter tobacco laws as compared with Texas [23,24]. Ethics approval was obtained from the respective institutional review boards (IRBs) of Harvard University, Baylor College of Medicine, and the University of Saskatchewan after extensive review, which ensured that adequate layers of protection were in place for our participants. Upon receiving the IRB approvals, we recruited 95 participants (smokers and nonsmokers) aged 18 to 34 years who resided in different cities within Massachusetts and Texas to participate in our 8-week smartphone tobacco tracking study.

Participants were required to meet the following criteria: (1) existing Android smartphone users with a data plan (although we covered the costs of their plan for the duration of the study) or would be willing to change their primary phone to a study-compatible phone, (2) consented to download a location-tracking smartphone app called *Ethica* and to keep their location-tracking feature switched on for the duration of the study, and (3) were willing to complete a pretest at the start of the study and a posttest at the end of the study as well as respond to EMAs that would be pushed to them. Ethica is a smartphone app designed to collect sensor-based data (eg, geolocations, accelerometry, and electrodermal activity) as well as contextual self-reports (eg, EMAs).

Once the participants downloaded Ethica (assisted by study staff) and registered using their email and a password, the study staff helped ensure that the location-tracking feature on their phones was enabled. The participants were then asked to complete the pretest via the app on their smartphone. This pretest contained questions pertaining to demographics, smoking status, number of cigarettes smoked in the past 30 days, and other related smoking attitudinal and behavioral questions. The location-tracking app began collecting geolocation data once registration was complete. At the end of the study, the participants completed a similar posttest.

#### Profile of Participants

Among the 95 participants (49 females, 42 males, 4 nonresponse), 51 were from Massachusetts and 43 were from Texas, with 1 nonresponse. Of all the 95 participants, when asked if they were of Hispanic, Latino, or Spanish origin, 54 (57%) reported they were “not of Hispanic, Latino, or Spanish origin”; 7 (7%) were “Mexican, Mexican American, Chicano”; 10 (11%) were “Puerto Rican”; 12 (13%) were “Dominican”; 1 (1%) was “Cuban”; 6 (6%) were “another Hispanic, Latino, or Spanish origin (eg, Guatemalan, Salvadoran, Honduran, Nicaraguan, Panamanian, Colombian, Venezuelan, Peruvian)”; and 5 (5%) with no response. In terms of race, 37/95 (39%) of our participants identified as “Black or African American”, 33/95 (35%) as “White”, and the rest identified themselves as a combination of different ethnic groups (eg, “American Indian or Alaska Native”, “Asian”, “Native Hawaiian”, or “other Pacific Islander”).

The median total combined household income was between US $20,000 and US $29,000 (from 1 [˂US $10,000] to 9 [≥US $75,000]; median 3.00 [US $20,000 and US $29,000]; SD 2.11), and the median education status was having *some college* (1 [completed grade school or less] to 8 [completed graduate or professional school after college]; median 5.00 [some college]; SD 1.32). In total, 53 participants self-identified as smokers, whereas 41 were nonsmokers, with 1 nonresponse. The participants were also asked to report the total number of cigarettes they smoked in the past 30 days (mean 11.3, SD 13.4).

### Data Management and Processing

#### Geolocations of Tobacco Retail Outlets

The geolocations of TROs were obtained from the North American Industry Classification System (NAICS). Developed by the Office of Management and Budget, NAICS is the federal standard business classification system based on the primary activities of businesses. We identified and extracted records for 252 TROs in Massachusetts and 1422 in Texas, based on the NAICS classification of *Tobacco Stores*. For this study, we chose to focus our analysis on TROs that were solely cigars, cigarettes, and tobacco dealers and retailers or smoke shops (NAICS8 code: Tobacco Stores) and excluded retailers whose primary descriptions were not in the area of tobacco sales (eg, beer, wine, and liquor stores; convenience stores; and gasoline stations with convenience stores), even though they might sell tobacco products. The reason for excluding these stores was that people might pass by or linger at these places because of reasons (eg, when shopping for groceries) other than tobacco purchase and consumption.

#### Participants’ Geolocations

Ethica collected approximately 31 million time-stamped geolocations from all the participants recorded in millisecond intervals. To increase the reliability of the data (eg, as there were multiple geolocations of individuals recorded when they were stationary), we collapsed participants’ geolocations into multiple 10-min time intervals and extracted the most accurate and representative longitude and latitude locations for each interval. In total, there were 279,840 geolocations of participants from Massachusetts and 227,991 geolocations for participants in Texas.

#### Geolocations of Smoking Behaviors

Questions regarding smoking behaviors were randomly administered 4 times a day to smokers via Ethica (between 8 AM and 9 PM on weekdays and between 10 AM and 9 PM on weekends). The geolocations of participants when they were smoking were captured from their responses to the question “Have you smoked in the past hour,” in which they were asked to select from the following responses: (1) I smoked a cigarette in the past hour, (2) I smoked an electronic cigarette (e-cigarette) in the past hour, (3) I used another tobacco product in the past hour, (4) I am smoking a cigarette right now, (5) I am smoking an e-cigarette right now, (6) I am using another tobacco product right now, and (7) I have not smoked. To obtain the geolocations of the participants when they were smoking, we extracted the longitude and latitude of smokers at the time if they indicated that they were smoking a cigarette or an e-cigarette or using another tobacco product *right now.* A total of 10,393 smoking geolocations in Massachusetts and 10,187 in Texas were recorded.

#### Geolocations of Tobacco Message Exposure

Through Ethica, participants were able to take or upload photos of tobacco messages and advertisements they came across in their communities (eg, billboards, TROs) or on the internet. After this, they were prompted to answer an EMA survey where the participants were given the options to identify the messages as either antitobacco or protobacco and to report when they saw the message (where 1=I see it right now; 2=in the past hour; 3=in the past 1-5 hours; 4=more than 5 hours ago). The latitude and longitude of the photos and EMA surveys were logged using Ethica.

### Statistical Analysis

Data were imported into ArcMap 10.6.1 for mapping and statistical analyses, where we conducted *spatial autocorrelation* and *optimized hot spot analysis* as well as *power-law analysis* in R studio (version 1.8383) to address all the study objectives. The global spatial autocorrelation was used to test for the presence of spatial variation in a given dataset [25], specifically in examining the correlation among data points that are close to one another and to determine if there is a nonrandom spatial clustering among data points that were in close proximity [26]. The global Moran index is a statistic that indicates the presence of statistically significant spatial clustering, which produces a number between –1 and +1. A negative value indicates the presence of negative spatial autocorrelation, which is the tendency for dissimilar values to be located together. On the other hand, positive values indicate the presence of positive spatial autocorrelation, where data points with similar values are clustered together [25]. If the presence of spatial clustering was detected, we then conducted the optimized hot spot analysis to examine the locations of the *hot spots* and *cold spots* of TROs, smoking, and antitobacco messages.

The power-law analysis aimed to test if there was an observable power-law distribution in the data. A power-law distribution is also known as a heavy tail distribution, where smaller values on the *x*-axis correspond to large values in the *y*-axis. In other words, in the context of this study, if a power-law distribution is observable, a small number of TROs would attract the most human traffic. This may suggest that certain TROs are more popular, or centrally located, such that people are more likely to pass by as compared with TROs located in obscure locations.

## Results

**Figure 1:**
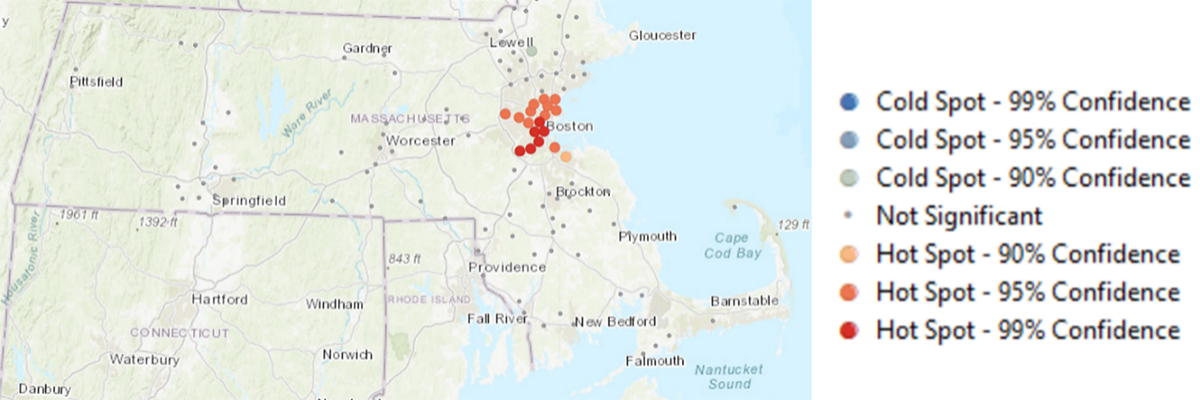
Hot spots of tobacco retail outlets in the state of Massachusetts.

**Figure 2:**
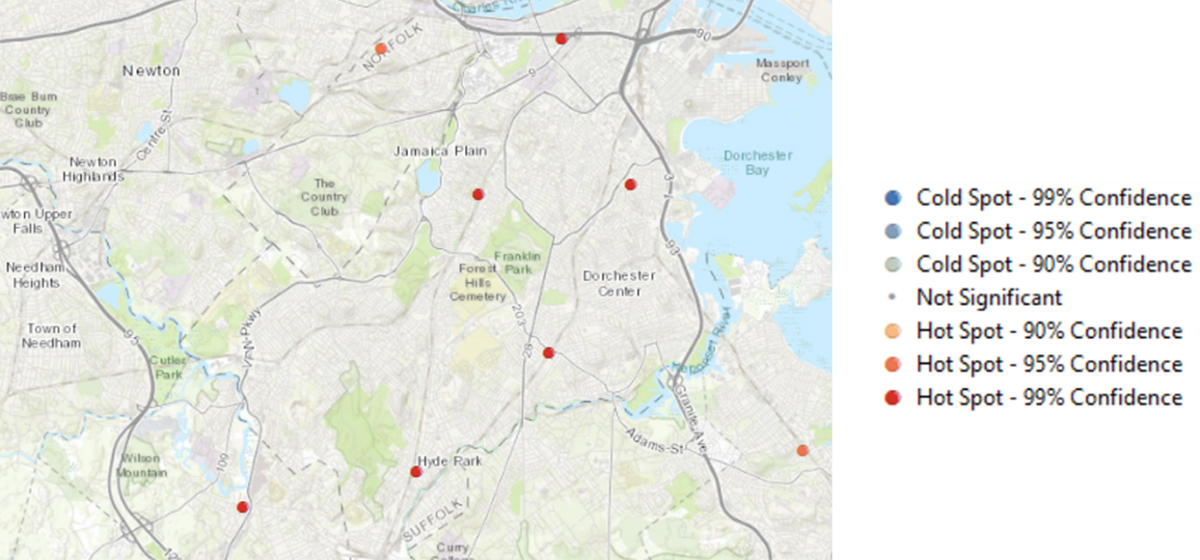
Zoomed-in view of the most significant hot spots in the city of Boston.

**Figure 3:**
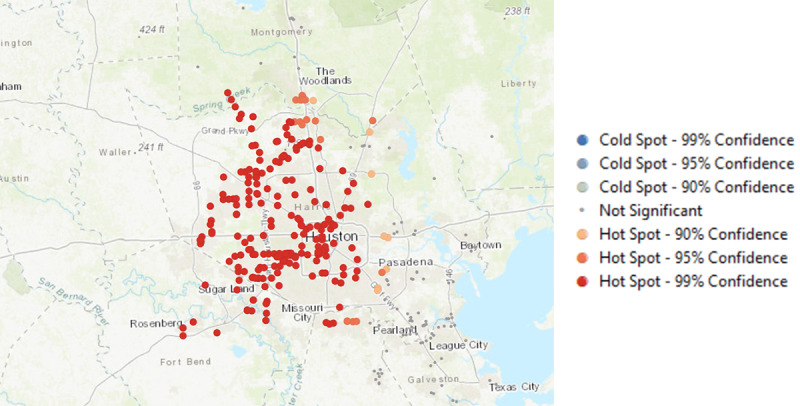
Hot spots of tobacco retail outlets in the state of Texas.

Our first objective was to identify if there were concentrations of TROs (ie, TRO hot spots) in Massachusetts and Texas (see Figures 1-3). To do so, we conducted spatial autocorrelation on the geolocations of TROs and determined if there was a statistically significant spatial clustering of TROs. The results suggest that there was evidence of clustering of TROs in both Massachusetts (global Moran index=0.79; *z*=8.04; *P*<.001) and Texas (global Moran index=0.69; *z*=5.85; *P*<.001). Next, optimized hot spot analysis in ArcMap showed that there was a statistically significant clustering of TROs in the city of Boston, with the most significant clusters found in Dorchester Center, Jamaica Plain, and Hyde Park (*z*≥3.50; *P*<.001). The TRO hot spots in Texas were found in Houston, and they were in places such as Spring, Jersey Village, Bunker Hill Village, Sugar Land, and Missouri City.

Our second objective was to draw upon both passive (ie, geolocations) and active (ie, self-reports) data to identify (1) the most popular TROs, (2) the areas in which participants were most likely to smoke, and (3) the locations where participants reported being exposed to tobacco messages and where these concentrations of protobacco and antitobacco messages were.

**Figure 4:**
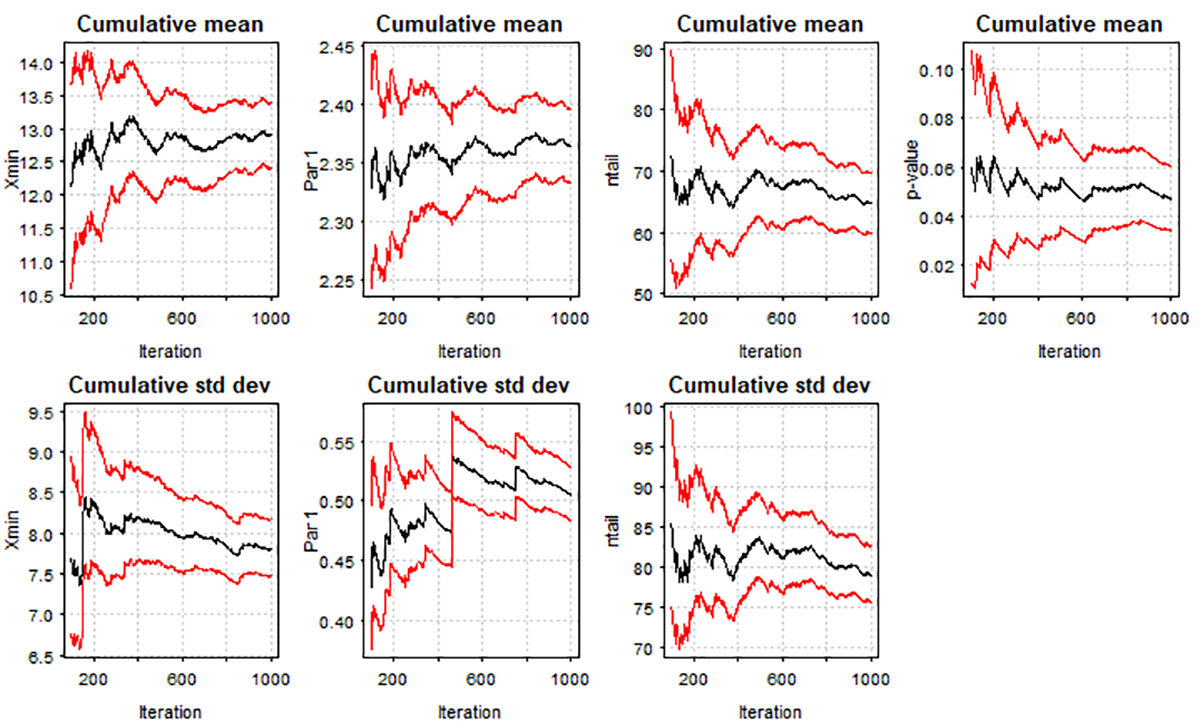
Cumulative mean of power-law analysis for traffic of tobacco retail outlets in Massachusetts.

To examine which were the most popular TROs—if a small number of TROs attracted the most traffic—we tested if a power-law distribution was observable by analyzing geolocations of participants near the TROs. To do so, we created a 100-m buffer around all the TROs in our dataset and performed a spatial join with all the geolocations that intersected within the buffer. The selection of a 100-m buffer was consistent with previous research [27]. We then exported the data to R studio to fit a power-law distribution in accordance with the steps recommended by Clauset et al [28]: (1) construct a discrete power-law distribution object, (2) estimate the *x*_min_ and exponent *α* of the power law and assign them to the power-law object, and (3) bootstrap to obtain the *P* value for the hypothesis test of if the data followed a power-law distribution. In the Kolmogorov-Smirnov test, the null hypothesis is that observations will follow a specific distribution, whereas the alternative hypothesis specifies that a set of distribution does not follow a specific distribution. As such, to claim that observations follow a power law distribution, the *P* value would have to be equal or more than .05 for the null hypothesis to be accepted, thereby indicating the presence of a power-law distribution. The analysis found marginal support for the power-law distribution (*D*=0.12; *P*=.05) for TROs in Massachusetts (Figure 4) but not for Texas. The top TROs that attracted the most human traffic from our sample were in the neighborhood of Roxbury in the city of Boston and Methuen, a city close to Boston.

**Figure 5:**
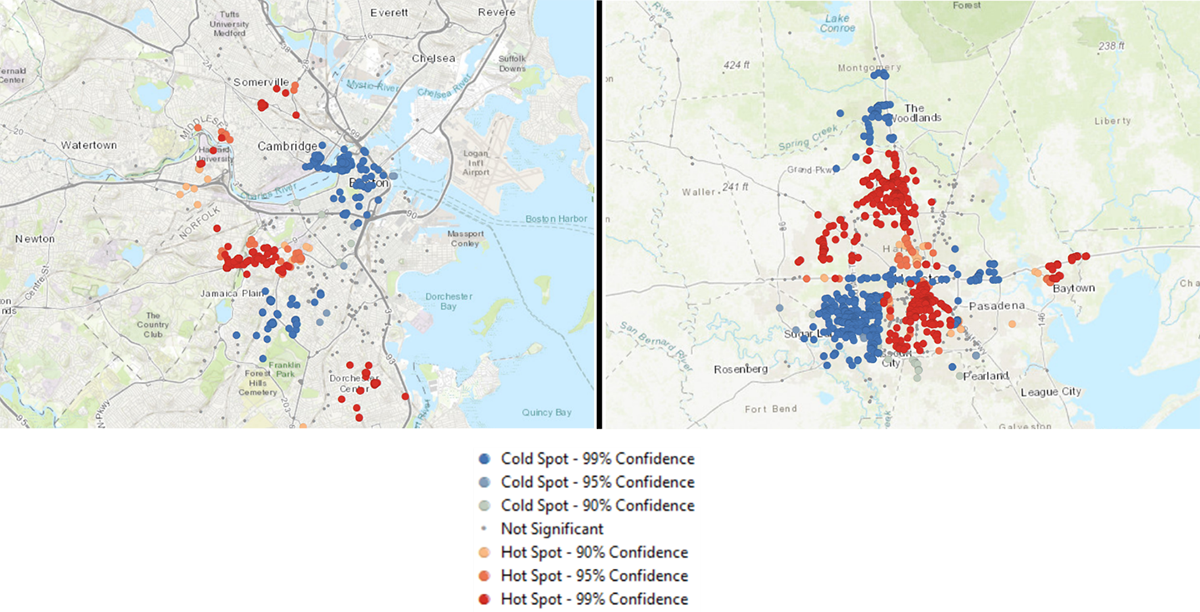
Clusters of smoking hot spots in Massachusetts (left) and Texas (right).

Next, to identify the areas where participants were most likely to smoke (ie, smoking hot spots), we conducted spatial autocorrelation on geolocations where the participants reported their smoking behavior through the EMAs and determined if there was a statistically significant spatial clustering of smokers who reported smoking (Figure 5). The results suggest that there was significant clustering in both Massachusetts (global Moran index=0.29; *z*=34; *P*<.001) and Texas (global Moran index=0.25; *z*=63.5; *P*<.001). Next, we conducted optimized hot spot analysis, and the results showed that in Massachusetts, the heaviest smokers (based on the number of cigarettes smoked in the past 30 days) tended to report that they smoked in Dorchester Center, Roxbury Crossing, Lawrence, and Peabody (*z*≥2.84; *P*<.001). In Texas, the heaviest smokers tended to report smoking in West University Place, Aldine, Jersey Village, Spring, and Baytown (*z*≥2.67; *P*<.001).

To identify the locations where the participants reported being exposed to antitobacco and protobacco messages, we examined the photos taken by the participants through the app where they rated if the messages were either antitobacco or protobacco. In Massachusetts, there were 41 antitobacco and 48 protobacco messages reported (see Multimedia Appendix 1). The top 3 most frequent platforms for exposure to antitobacco messages in Massachusetts were on (1) television and others (19.5% each, 8/41 for television and 8/41 for others), (2) store sign or display (7/41, 17.1%), and (3) billboard/bus/train stop advertisements (6/41, 14.6%). The top 3 highest exposures to protobacco messages were on (1) store sign or display (27/48, 56.3%), (2) newspaper or magazine (7/48, 14.6%), and (3) website (5/48, 10.4%).

In Texas, there were 63 antitobacco and 43 protobacco messages (Multimedia Appendix 1). The top three highest exposures to antitobacco messages in Texas were (1) others (21/63, 33.3%), (2) television (14/63, 22.2%), and (3) store sign or display (7/63, 11.1%). The top 3 highest exposures to protobacco messages were on (1) store sign or display (25/43, 58.1%), (2) others (7/43, 16.3%), and (3) television (4/43, 9.3%).

**Figure 6:**
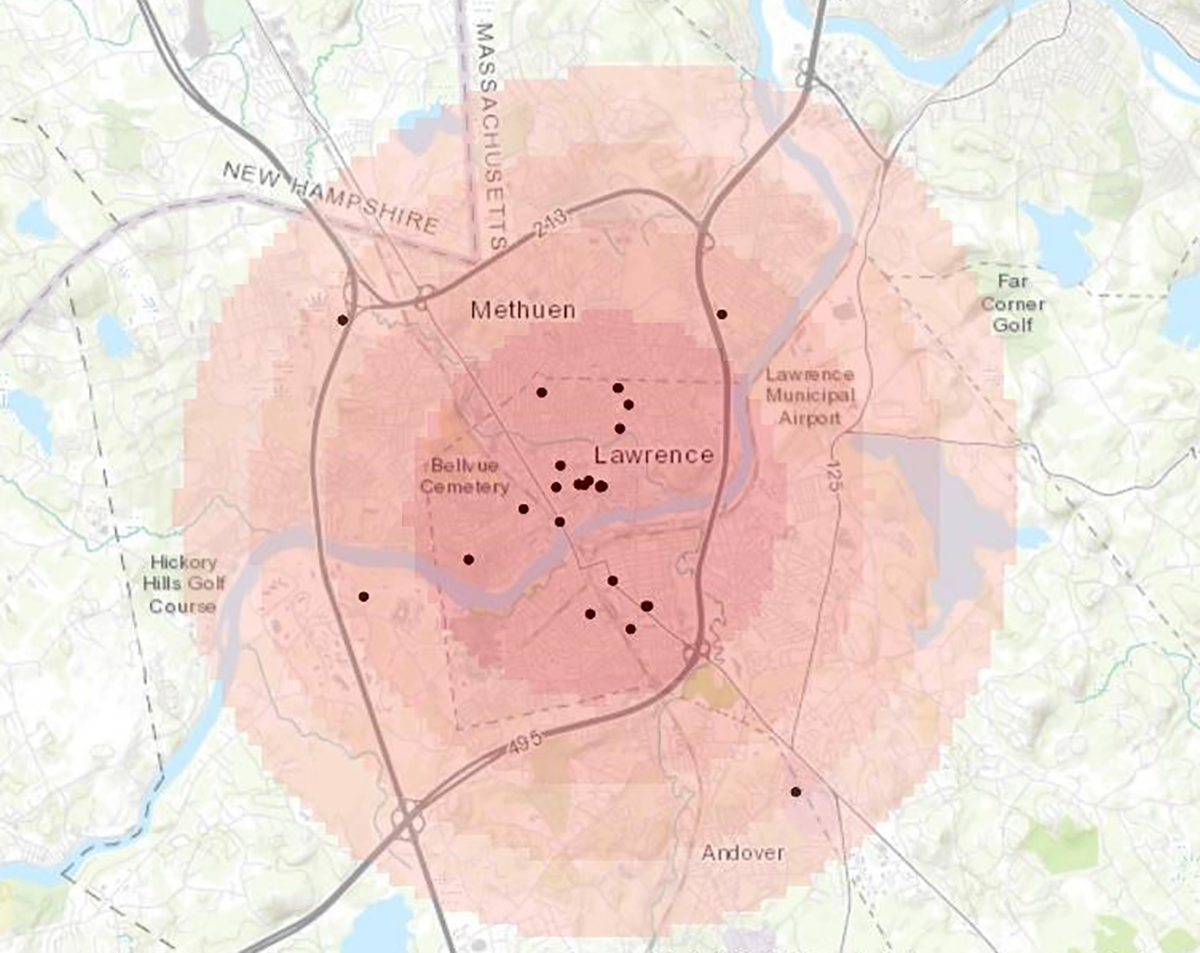
Clusters of reported antitobacco messages in Lawrence, Massachusetts.

Finally, we aimed to examine if there were spatial clustering of tobacco messages and if such clusters were located near TROs or smoking hot spots. We analyzed the data using spatial autocorrelation, and the results suggested that there was evidence of antitobacco message clustering in Massachusetts (global Moran index=0.28; *z*=1.89; *P*=.06) but not in Texas (global Moran index=–0.12; *z*=0.73; *P*=.07). We then conducted an optimized hot spot analysis for antitobacco messages in Massachusetts, and the results showed that the clustering of antitobacco messages (*z*=3.85; *P*<.001) only occurred in Lawrence in Massachusetts (Figure 6). There was no evidence of protobacco message clusters.

## Discussion

This study showcases how data from smartphone apps could significantly inform tobacco control communication efforts when used to complement existing data sources, such as the geolocations of TROs in our study obtained from NAICS [29]. Using these methods, we were able to identify the locations where our participants were exposed to tobacco messages (anti- and pro-), the specific TROs that attract the highest level of patrons, as well as areas where individuals were most likely to smoke. There are several notable findings from our results. The data showed that physical locations still matter more than online tobacco messages when considering where people were most likely to encounter external cues for tobacco use, such as locations of TROs and areas where protobacco messaging were reported. From NAICS data, there was a concentration of TROs in economically disadvantaged areas within Boston, such as the Dorchester Center and Jamaica Plain [30]. In Texas, our data showed that there was a high concentration of TROs across Houston.

The data from smartphones complement traditional tobacco surveillance data, such as population health surveys [31], in that smartphone data provide context to how access to tobacco products and exposure to marketing and promotional efforts may influence tobacco use behavior within underserved communities. For example, we found that in both Massachusetts and Texas, participants reported that they predominantly encountered protobacco messages at store signs or displays as compared with web-based sources. This is somewhat surprising considering the increasing concern about the influence of social media posts in the promotion of tobacco, either through user-generated content on social media or through targeted industry web-based advertising efforts [32,33]. In contrast, although our participants reported encountering antitobacco messages through web-based and offline sources, they were most likely to come across antitobacco messages on television. In addition to airing messages on mainstream media, public health officials should consider boosting efforts in placing antitobacco messages around TROs.

Another finding is that in both Massachusetts and Texas, participants reported encountering fewer antitobacco messages in newspapers or magazines as compared with protobacco messages. This is consistent with the findings from a recent study [34], which aimed to determine the extent of exposure to federal court-ordered antismoking advertisements—where tobacco companies were required to pay for these advertisements to correct smoking misinformation [35]—among a nationally representative sample of the adult population in the United States in 2018. The study found that the overall estimated exposure to antismoking advertisements was generally low (40.6%), with the lowest exposure rates found among people aged 18 to 34 years (37.4%), those who had high school education or less (34.5%), those who earned less than US $35,000 annually (37.5%), and Hispanic smokers (42.2%). Although it was difficult to definitively pinpoint why our participants reported low exposure to antitobacco advertisements in newspapers, one plausible reason was that young people such as those in our sample may not be using print newspapers and magazines as much as the internet and social media [36,37], and thus, they would be less likely to come across antitobacco messages across traditional media platforms. In addition, research has documented that people from underserved communities were less likely to use newspapers as their primary news sources as compared with individuals from higher SEPs [38].

Second, this type of smartphone data collection allows one to target strategic areas for antitobacco message placement. For instance, in the state of Massachusetts, there was evidence of antitobacco messages only in Lawrence, which traditionally has a higher percentage of adult smokers and TROs per 1000 adults as compared with other parts of Massachusetts [39]. Although this was a positive step, there was a need for broader dissemination of antitobacco messages to reach other areas where popular TRO hot spots were found (Dorchester Center, Jamaica Plain, and Hyde Park), specific TROs (Roxbury and Methuen) with highest human traffic, as well as areas where smoking was concentrated (Dorchester Center, Roxbury Crossing, and Peabody).

Third, it is evident that the use of smartphone data to inform antitobacco messaging efforts for underserved communities is not a magic pill solution, as it would need concurrent *supply side* tobacco control regulations to be most effective. In Houston, the widespread prevalence of TROs remained problematic for targeted antitobacco messaging to be efficacious. In other words, effective and targeted antitobacco messaging in Texas would need to be accompanied by concurrent supply side solutions, such as restricting the number of TROs or increasing tobacco taxes.

Despite the study’s significant strengths, there are limitations. First, we relied on a small sample of individuals from underserved communities, and the results would not be generalizable to the overall population. For example, the locations of popular smoking areas could be heavily influenced by the characteristics of our sample. Second, as in all studies that employ smartphone apps, the geolocations were only captured when the smartphones were operational. Third, this methodology does not guarantee that exposure to all antitobacco messages is captured. Participants might not be able to snap a picture of the antitobacco message on a billboard in time if they were driving or traveling in a car. Finally, we recognized that, similar to many smartphone tracking studies, there are issues pertaining to privacy because of the amount of data collected that may not relate directly to the study’s objectives. Considering that we were working with underserved communities that were arguably more vulnerable than the general population, we prioritized the privacy protection of participants from the beginning of this study and took significant steps in communicating with our participants the privacy protection measures we have implemented.

At the policy and system architecture level, Ethica was built to be compliant with the General Data Protection Regulation requirements, which extended data protection for different types of health data collected from individuals [18,40]. In other words, our participants had the right to access and delete their own data. If the participants did not have the technical skills to do so, Ethica would provide technical support as needed. In addition, Ethica allowed the participants to request a copy of their data, and the support staff would provide them with a machine-readable file containing all the data collected about them. On a practical level, Ethica was designed in such a way that participants could snooze their study participation for some time. For instance, there was an incognito function where participants could pause data collection (eg, tracking of their geolocations) at any time they wanted.

Despite these limitations, this study presents a novel way of integrating passive and active data from smartphones with traditional tobacco surveillance information to help inform tobacco control efforts within underserved communities. We recommend that public health researchers continue to explore how to capitalize on big data from smartphones for tobacco control. For instance, future studies could extend our study by recruiting a larger sample of participants from different states and examining how fluctuations in emotions (captured by the EMA) could play a role in influencing tobacco use. Future research could also design smartphone-based interventions examining the optimal locations and time to administer antitobacco messages to people from underserved communities. In conclusion, smartphone data can inform tobacco control efforts in a powerful way, and health organizations and public health researchers should take advantage of this data revolution to strengthen tobacco control efforts to benefit the health of underserved communities [20].

## Appendix

Multimedia Appendix 1 Tobacco messages reported in Massachusetts (Table 1) and Texas (Table 2).

## Abbreviations

e-cigarette: electronic cigarette
GED: General Education Diploma
IRB: institutional review board
NAICS: North American Industry Classification System
SEP: socioeconomic position
TRO: tobacco retail outlet
