# Supplementary material for: The Potential of Smartphone Apps in Informing Protobacco and Antitobacco Messaging Efforts Among Underserved Communities: Longitudinal Observational Study
Source: J Med Internet Res. 2020 Jul 7;22(7):e17451. doi: 10.2196/17451 (PMC7381035; doi:10.2196/17451)
Supplement: Multimedia Appendix 1 [file jmir_v22i7e17451_app1.docx]

| **Table 1. Tobacco messages reported in Massachusetts** | | | | |
| --- | --- | --- | --- | --- |
| **Location of tobacco messages** | **No. of antitobacco messages (n = 41)** | **Proportion of all antitobacco messages (%)** | **No. of protobacco messages (n = 48)** | **Proportion of all protobacco messages (%)** |
| Newspaper or magazine | 3 | 7.3% | 7 | 14.6% |
| Store sign or display | 7 | 17.1% | 27 | 56.3% |
| Billboard/bus/train stop advertisements | 6 | 14.6% | 0 | 0.0% |
| Website | 4 | 9.8% | 5 | 10.4% |
| Email | 0 | 0.0% | 1 | 2.1% |
| Television | 8 | 19.5% | 4 | 8.3% |
| Bar or restaurant | 0 | 0.0% | 0 | 0.0% |
| App | 1 | 2.4% | 1 | 2.1% |
| Social media | 3 | 7.3% | 1 | 2.1% |
| Others | 8 | 19.5% | 2 | 4.2% |
| Never answered | 1 | 2.4% | 0 | 0.0% |
|  |  |  |  |  |
|  |  |  |  |  |
| **Table 2. Tobacco messages reported in Texas** | | | | |
| **Location of tobacco messages** | **No. of antitobacco messages (n = 63)** | **Proportion of all antitobacco messages (%)** | **No. of protobacco messages (n = 43)** | **Proportion of all protobacco messages (%)** |
| Newspaper or magazine | 0 | 0.0% | 1 | 2.3% |
| Store sign or display | 7 | 11.1% | 25 | 58.1% |
| Billboard/bus/train stop advertisements | 1 | 1.6% | 1 | 2.3% |
| Website | 4 | 6.3% | 1 | 2.3% |
| Email | 1 | 1.6% | 0 | 0.0% |
| Television | 14 | 22.2% | 4 | 9.3% |
| Bar or restaurant | 5 | 7.9% | 3 | 7.0% |
| App | 5 | 7.9% | 0 | 0.0% |
| Social media | 3 | 4.8% | 1 | 2.3% |
| Others | 21 | 33.3% | 7 | 16.3% |
| Never answered | 2 | 3.2% | 0 | 0.0% |
